# COVID‐19 as moral breakdown: Entangled ethical demands experienced by hospital‐based nurses in the early onset of the pandemic

**DOI:** 10.1111/nin.12508

**Published:** 2022-06-16

**Authors:** Caroline Trillingsgaard Mejdahl, Berit Kjærside Nielsen, Mimi Yung Mehlsen, Maj Rafn Hollesen, Mathilde Zilén Pedersen, Georgij Engkjær‐Trautwein, Louise Vase Funch, Morten Deleuran Terkildsen

**Affiliations:** ^1^ DEFACTUM—Public Health & Health Services Research Aarhus Denmark; ^2^ Department of Psychology and Behavioural Sciences Aarhus University Aarhus Denmark; ^3^ Department of Forensic Psychiatry Aarhus University Hospital Psychiatry Aarhus Denmark

**Keywords:** COVID‐19 pandemic, ethics, nurses, nursing ethics, qualitative study, thematic analysis

## Abstract

2020 saw the rapid onset of a global pandemic caused by the SARS‐CoV‐2 virus. For healthcare systems worldwide, the pandemic called upon quick organization ensuring treatment and containment measures for the new virus disease. Nurses were seen as constituting a vital instrumental professional component in this study. Due to the pandemic's unpredictable and potentially dangerous nature, nurses have faced unprecedented risks and challenges. Based on interviews and free text comment from a survey, this study explores how ethical challenges related to “being a nurse” during the COVID‐19 pandemic was experienced and understood by Danish hospital‐based nurses. Departing from anthropologist Jarett Zigon's notion of moral breakdown, the study demonstrates how the rapid onset of the pandemic constitutes a moral breakdown raising ethical demands for nurses. Analytically we identify three different ethical demands experienced by the nurses. These ethical demands are *Nursing and societal ethical demands, Nursing and personal ethical demands*, and *Nursing and conflicting ethical demands*. These demands represent not only very different understandings of ethical demands but also different understandings of ethical acts that are seen as necessary to respond to these demands.

## 1 INTRODUCTION

The major health crises posed by the COVID‐19 pandemic have given rise to worldwide attention to the performance of health professionals. Policymakers and public media praised nurses for their efforts in preventing the spread of the virus. Moreover, nurses were often presented as a crucial component in the successful care and treatment of COVID‐19 patients. This heavy public focus upon nurses during the COVID‐19 sparked salient interest in the role and responsibilities of nurses in times of pandemics. Because of the unpredictable nature of the pandemic, hospital‐based nurses have faced unprecedented risks and challenges. This study offers an in‐depth understanding of the ethical demands that nurses have felt called upon during the COVID‐19 pandemic.

### 1.1 Nursing in the light of COVID‐19

In December 2019 in Wuhan, China, the severe coronavirus disease caused by SARS‐CoV‐2 was discovered (COVID‐19), and in March 2020, The World Health Organization declared COVID‐19 a pandemic (WHO, 2020). Previous studies have established that health professionals are particularly vulnerable to developing adverse psychological reactions due to high levels of unfamiliarity and uncontrollability along with circumstances such as increased workload, lack of protective equipment, risk of infection, and ethical issues during pandemics (Liu et al., 2012; Lung et al., 2009; Wu et al., 2009). A growing body of literature documents severe degrees of harmful psychological symptoms amongst health professionals during the first wave of COVID‐19 (Luo et al., 2020; Vizheh et al., 2020). Research further recognizes that the nursing profession has demonstrated the most significant incidence of adverse psychological reactions compared to other professions (Luo et al., 2020; Pappa et al., 2020; Sanghera et al., 2020; Vizheh et al., 2020). The increased incidence of adverse psychological reactions among nurses may originate in the extraordinary efforts that have been demanded by the nursing profession, for example, a perceived expectation to neglect one's safety in the work of ensuring the health of the population under challenging work conditions (Boulton et al., 2021). Several studies have been published exploring the lived experiences of nurses during the pandemic, documenting sustained mental and emotional distress (Dagyaran et al., 2021; Iheduru‐Anderson, 2021; Marey‐Sarwan et al., 2021). A systematic qualitative review of psychosocial experiences of nurses during the COVID‐19 pandemic found that the pandemic had generated multiple challenges to the nursing practice, and frontline nurses experienced psychological, social, and emotional distress in coping with work, social relationships, and their personal life (Xu et al., 2021).

Recent studies further suggest that it is not only due to the increased risk of infection and exhausting work conditions that the nurses have experienced emotional distress during the pandemic (Gebreheat & Teame, 2021; Hossain & Clatty, 2021; Marey‐Sarwan et al., 2021; McConnell, 2020; Morley et al., 2020). A growing body of literature has shed light on the various ethical challenges nurses face during the pandemic (Gebreheat & Teame, 2021; McConnell, 2020; Morley et al., 2020; Peter et al., 2022). Morley et al. (2020) point to three overarching ethical issues experienced by nurses: the allocation of scarce resources; the safety of nurses, patients, colleagues, and families; and the changing nature of the patient‐nurse relationship. Further, a scoping review displayed similar conclusions, stating ethical challenges for nurses during the pandemic as related to lack of clinical, financial, informational, and supportive resources (Peter et al., 2022). Understanding the many ethical challenges nurses faced during the pandemic is fundamental to fully grasp the pandemics' complex and far‐reaching impact on the nursing profession. Such insight is critical to inform and develop the ongoing emotional, psychological, and practice support of nurses during and after the pandemic.

Less attention has been given to elevate discussions on nurses' ethical challenges on a more theoretical level. To understand nurses' ethical challenges on a conceptual level we applied Jarett Zigon's theoretical framework of “moral breakdowns”. Thus, departing from Zigon's notions on ethics and moral this study aimed to explore how ethical challenges related to working as a nurse during the first wave of the COVID‐19 pandemic were experienced and understood by Danish hospital‐based nurses.

## 2 METHODS

The study is a thematic analysis of qualitative data consisting of semi‐structured interviews supplemented with free text comments from a web‐based survey. We applied an interpretive methodological approach to analysis inspired by Braun and Clark (Braun & Clarke, 2006).

### 2.1 Ethical considerations

According to Danish law, this kind of qualitative study does not require notification to the Committee on Biomedical Research Ethics. All procedures performed in studies involving human participants followed the ethical standards of the institutional and national research committee and with the 1964 Helsinki declaration and its later amendments or comparable ethical standards. Informed consent was obtained, and all general requirements for health science research were followed.

### 2.2 Data collection

#### 2.2.1 Free text comments

Before the semi‐structured interviews, the participant took part in a web‐based survey launched on May 18, 2020, through social media. In the survey, we invited all hospital‐based nurses to participate in a study of nurses' mental health and well‐being. To be included in the survey, the participants were required to be a registered nurse currently working at a Danish hospital with sufficient mastery of Danish to complete a questionnaire (Mejdahl et al., 2022). The survey's main aim was to examine mental health (stress, anxiety, depression, insomnia) and their associated factors among Danish nurses during the COVID‐19 pandemic. In the survey, we invited participants to describe how they experienced being a nurse during the pandemic in an unlimited text box (We report the data on the survey's quantitative results in a separate article). Out of the total sample's 1160 participants who answered the survey, 351 supplemented their response with a free text comment. These comments were nuanced and content‐rich and constituted a supplement to this article's empirical material. We exported all free‐text comments to a word document (61 A4 pages).

#### 2.2.2 Semi‐structured interviews

We identified participants for interviews via the web‐based survey. Participants were asked to indicate if they were interested in participating in a subsequent individual interview in the survey, and 293 participants consented to participate in an interview. We utilized purposive sampling to include nurses who could provide in‐depth and detail information about the topic under investigation. Further, we sought variation, and therefore we included nurses representing diversity in age, geographical workplace, seniority, and work function during the first wave of the pandemic, yielding an interview sample of 21 nurses. Interviews were continued until enough rich storied data were generated to inform a robust analysis and substantial understanding of nurses' ethical demands.

Because of COVID‐19 restrictions, the semi‐structured interviews were conducted by phone from August 2020 to November 2020. The interviews lasted 33–61 min (mean: 50 min) and were all recorded and transcribed verbatim. We developed an interview guide based on an initial analysis of the qualitative data from the survey and a review of relevant literature. The themes of the interviews were broad and covered: (1) fear and concerns, (2) nursing roles and responsibilities, (3) physical and mental health issues, (4) public and media views on nurses, (5) family life, and (6) organizational issues. Using general questions and prompts designed to release participant experiences, our research team invited nurses to share their personal experiences of being a nurse during the COVID‐19 pandemic.

### 2.3 Analysis process

We facilitated data management using the qualitative software program NVivo^TM^ (Qsr International, 2016). The first and last author performed the data analysis supported by discussions with the research team. We used a thematic analysis approach. Inspired by the procedure proposed by Braun and Clarke (Braun & Clarke, 2006) we initially read the transcribed interview data several times while noting initial ideas to become familiarized with the data. Interesting features across the data set were then systematically coded, and data relevant to each initial code were carefully collected. Having arranged our data in meaningful groups, we started identifying themes. At this stage, we found a strong resonance between our codes, our initial themes, and a theoretical framework (Terkildsen et al., 2021). Departing in Jarett Zigon's theoretical framework of “moral breakdowns,” we identified one overarching theme; nurses' ethical demands during a pandemic (Zigon, 2007). Based on Zigon's framework we re‐coded the material focusing on identifying moral breakdowns, ethical demands, and ethical responses. We then sorted the different initial codes into potential themes and collated all the relevant coded data extracted within the identified themes. At this point, we included the empirical data from the overall sample's free‐text comments in our analysis. Next, we moved on to reviewing and refining our proposed themes. Thus, when checking if our themes worked concerning both the coded extracts and the entire data set, we discovered that some of our different themes could be collapsed into one theme. Next, we defined and named the themes. Focusing on identifying the “essence” of each theme, we named the themes and identified sub‐themes. Finally, we selected compelling extract examples that captured the essence of themes and subthemes (Braun & Clarke, 2006).

### 2.4 Theoretical framework

This paper theoretically departs from anthropologist Jarett Zigon's (2007) notion of *moral breakdown*. According to Zigon (2008, 2009) morality may be conceptualized in three interrelated spheres: (1) the morality of institutions understood as particular kinds of morality produced and reproduced by formal and nonformal social organizations in society wielding different forms of power over individuals, (2) the public discourse of morality understood as the explicit public articulations of moral beliefs (such as in public media) not directly related to institutions and finally, and the main subject of this paper, (3) morality understood as the habitual construct in Zigon's own words understood as “the acquired attitudes, emotions and bodily dispositions of a person throughout their life” (Zigon, 2008; p. 17). Though articulations of the moral may be quite explicit in the spheres of institutions and public discourse Zigon draws upon Heidegger's famous hammer example to note that in many of lives ordeals, morality is an inherent disposition, and much like the everyday use of a hammer, we do not think of moral ways of being and doing social life. Just as carpenters do not reflect upon the nature of the hammer or the practice of using a hammer when hammering in everyday practice, so too is morality simply *ready‐to‐hand*, an extension of our social selves non‐noticeable when performed in everyday practices (Zigon, 2007). Morality in this sense should therefore not be understood as reducible to rules and principles laid out explicitly by the spheres of social institutions and public discourses but also as the guiding principles, habitual and, therefore, unreflective dispositions of everyday life attained over a lifetime of socially performed techniques yet constantly shaped and reshaped by the ongoing social experiences of person lives (Zigon, 2008). In practice, this morality allows us to nonconsciously act in ways that are acceptable to others without even considering our actions. In practice, it allows us to be social (Zigon, 2008; p. 164, see also Zigon, 2007; p. 135).

However, just as a hammer may break, making it reflexively *present‐to‐hand* to the carpenter calling him or her to question its form and function, so too are lives contingent. According to Zigon (2007, 2008), occasionally, events unfold that fundamentally make the *ready‐to‐hand* nature of our being‐in‐the‐world breakdown. In such instances, we become forced to reflect, deliberate, and respond to the problems or dilemmas raised by such events. These moments are moral breakdowns. According to Zigon, when they occur, we become called to act. These are ethical moments, and they bring together different spheres of morality from the social world around us, informing us and raising ethical demands that force us to act in ways allowing us to return to the unreflective moral dispositions of everyday life (Zigon, 2009). Here Zigon makes an essential distinction between morality and ethics. Where morality may be understood in terms of often unreflexive dispositions of social life, ethics is the reflective “stepping‐away” from these embodied dispositions. Ethics are the deliberations that moral breakdowns bring about. They are our responses, the actions and tactics we apply to the ethical demands we identify as brought about by the moral breakdown (Zigon, 2008).

## 3 FINDINGS

The interview sample comprised 21 registered nurses, 20 women and 1 man, aged 25–63 years. All participants worked in a Danish public hospital, and their experience in nursing practice ranged from 0.5 to 39 years (mean: 14.6) (Table 1). The free‐text sample comprised 351 registered nurses (Table 2).

**Table 1:** Interview participants' demographic characteristics

	*N* = 21 (%)
Sex	
Female	20 (95)
Male	1 (5)
Age (years)	
20–35	7 (33)
36–50	9 (43)
51–65	5 (24)
>65	0 (0)
Years of working as a nurse	
<3 years	6 (28)
3–10 years	3 (14)
11–20 years	6 (29)
>20 years	6 (29)
Region	
Capital	5 (24)
Central Denmark	7 (33)
North Denmark	2 (10)
Southern Denmark	3 (14)
Zealand	4 (19)
Department	
General medicine	5 (24)
Intensive care unit	2 (10)
Emergency/acute	5 (24)
Psychiatry	2 (10)
Outpatient clinic	6 (28)
Other	1 (4)
Caring for patients infected with COVID‐19	
Yes	14 (67)
No	7 (33)

**Table 2:** Free‐text participants' demographic characteristics

	*N* = 351 (%)
Sex	
Female	345 (98)
Male	6 (2)
Age (years)	
20–35	68 (20)
36–50	162 (46)
51–65	117 (33)
>65	3 (1)
Not registered	1 (0)
Years of working as a nurse	
<3 years	36 (10)
3–10 years	63 (18)
11–20 years	119 (34)
>20 years	133 (38)
Region	
Capital	72 (21)
Central Denmark	185 (53)
North Denmark	22 (6)
Southern Denmark	35 (10)
Zealand	36 (10)
Not registered	1 (0)
Department	
General medicine	57 (16)
Intensive care unit	18 (5)
Emergency/acute	20 (6)
Psychiatry	11 (3)
Pediatrics	11 (3)
Surgical	48 (14)
Perioperative care or surgical areas	34 (10)
Outpatient clinic	72 (21)
Anesthetics	29 (8)
Other	50 (14)
Not registered	1 (0)
Caring for patient infected with COVID‐19	
Yes	184 (53)
No	163 (46)
Not registered	4 (1)

Our analysis finds that understandings and experiences of being a nurse during COVID‐19 in Denmark were expressed as someone taking upon vital responsibilities. Among the nurses, a strong sense of agreement was found that the COVID‐19 pandemic could be seen as a kind of “significant event” raising pertinent stakes that demanded specific actions to be taken as a response. However, despite sharing overall ideas of COVID‐19 being a “significant event,” we found that the nurses in our study framed the stakes raised by the pandemic very differently and, in turn, presented very different understandings of needed responses.

### 3.1 To be a nurse, Covid‐19, and dangers (moral breakdown)

In the interviews, when asked to describe how they had experienced the early stages of the pandemic, we found strong resonance between the nurses' descriptions. When first hearing about COVID‐19, nurses explicated how they felt, and it felt as if standing in the middle of a significant event in history: One nurse uttered:

Well, I actually think it was kind of surreal to be a part of such a historic event somehow. I've never experienced anything like this before, and I do not think I ever will again.

Despite, as seen in the above quote, being presented as something surreal, the interviews also displayed significant agreement among the nurses regarding the potential seriousness of the situation. Having not yet arrived in Denmark, several nurses emphasized how the daily TV broadcasts from around the world (at that time notably from Italy) and the still uncertain nature of the disease made them ponder the potential stakes and dangers that could fall upon them as nurses if or when the pandemic reached Denmark. One nurse uttered during an interview:

I didn't know what was going to happen. No, not at all. We did not really know what it was either. Would it be like in Bergamo (Italy) in a month? It could just as well have been like that. In the beginning, we saw those broadcasts from Italy, which were not very far away after all.

Our material revealed that the pandemic's uncertain but potentially dangerous nature brought a level of disruption for the nurses – a breakdown in which the everyday un‐reflected moral dispositions tied to their idea of being nurses was suddenly brought out in the open and made the subject of reflexive ethical questioning. What would it mean for the Danish nurses to be nurses during an impending pandemic? What could be the possible consequences for their everyday lives, and how would they act accordingly? These were salient questions raised in many interviews by the nurses. As an example, one nurse said:

We were constantly informed about new restrictions and given new guidelines we had to follow. We got all this information about the situation in Italy. So it was with a certain amount of nervousness and anxiety, we faced the pandemic. Asking ourselves; what will happen in Denmark? Is the situation going to be as bad as in Italy? Are we going to be able to keep up? Do I have to work around the clock? How do I even manage my family in this situation?

However, where the nurse broadly agreed that the encroaching pandemic had made them reflexively inquisitive towards the potential ethical stakes and demands facing them, what they saw as ethical stakes and demands, and towards whom these ethical stakes and demands pertained differed highly among them. In the material, we identified three very different ethical demands seen by the nurses as emerging after the moral breakdown brought on by the COVID‐19 pandemic in Denmark. *Nursing and societal ethical demands, Nursing and personal ethical demands, and Nursing and conflicting ethical demands*. With these three very different understandings of ethical demands, we find very different understandings of ethical acts that are seen as necessary to respond to these ethical demands.

### 3.2 Nursing and societal ethical demands

Several nurses expressed that the breakout of the COVID‐19 pandemic constituted a particular kind of threat to society as a whole – a society to which these nurses saw themselves as an inherent part. Several expressed the arrival of COVID‐19 as reminiscent of a wartime crisis, and in these descriptions, the nurses often presented themselves as a group called together for a common societal cause. One nurse talked about her role as a nurse in society and described COVID‐19 as a defining moment calling upon her and her colleagues as a nursing group to step up and fulfill a collective duty in times of need:

It's a duty. But it's not like I'm just doing it because I have to do it. I do it because I need to do this. We need to step up. We were so ready, and everyone geared up. We were so ready, like being ready for a war.

Another nurse further elaborated how this feeling of collective duty made her feel pride to be a professional capable of serving society as a nurse:

Well, I actually think it was cool to help contribute to society that way (…). I felt that now it was my chance to be the one who could make a difference, even more than I already do in my work.

Strong feelings of unity were salient in interviews, and fellow nurses expressed their feelings of explicit ethical demands to act in ways beneficial to other nurses. One nurse expressed feeling guilty toward nursing colleagues at nearby hospitals who were caring for many COVID‐19 patients: “*You feel kind of guilty when you hear about your colleagues at others hospitals that are extremely busy, and you are not busy yourself*.” In this expression, in which an ethical demand to act diligently so that other nurses would not find themselves faced by pressure at one's expense was unfolded, ethical demands towards patients were also explicitly framed.

One nurse explained how being a nurse implied a particular societal duty to ensure the safety of patients even when faced with the potential dangers of a new pandemic:

We have a responsibility to care for these patients. We have a responsibility to act under the conditions society provides for us. That's how it will always be. I have an obligation. I cannot just pack my bags and leave if I think the patients are too sick. So, yes, I have an extraordinary responsibility to do my job under the conditions given to me.

Another nurse expressed how her greatest fear and sense of urgency when facing the pandemic had been the consequences it may have for patients in Denmark. Seeing the pictures from hospital wards in Italy and hearing about shortages in staff and materials had filled her with anxiety and fear that she would have to act as a judge saving the lives of some patients at the expense of others. A situation speaking against what she saw as the ideals of being a nurse:

What I feared the most was, of course, that it would be like the situation in Italy and that we would be forced to give priority to some patients. I feared that we would have to say to a 60‐year‐old patient in need of a respirator: “unfortunately, we need to give the respirator to a 45‐year‐old, so you have to do without.”

For these nurses, their feelings of ethical demands to care for patients were expressed as overriding their concerns about the dangers that the pandemic could have for them on a personal level. A nurse said:

I have not been afraid of being infected myself. What scared me was the thought of what would happen if many of us nurses became infected; who should then take care of all the patients? (…). I believe that as a nurse, you have a special responsibility to care for patients and help where it is needed.

Another nurse emphasized the deep‐founded nature of this ethical demand, expressing how the duty to serve society and its patients as nurses extended beyond individuals' needs:

Of course, you have a special responsibility as a nurse. Imagine if they asked us to go and care for patients in the covid‐department, and we all declined. That just wouldn't be okay. Of course, you have a responsibility to help where needed.

Typical of these understandings was the idea that nurses not only belonged to society but also presented themselves as representing a particular kind of professional group that, via their work in the public health care system, felt a specific ethical demand to ensure the protection and survival of this society now faced by a threat of potential destabilization caused by the pandemic. One nurse remarked:

I believed I had the skills, so I simply had to participate and care for these patients. To me, it comes with the profession. When there is such a crisis in your country, you have to step up. Who should otherwise do it?

Though these nurses often explicitly connected engagement in care‐work in the public health care system with their fulfillment of a moral obligation to society, some also framed their moral obligation to society as a task of transgression from health care to society as a whole. One nurse remarked: “*So, both the responsibility you've had at work in caring for these patients, but I also believe that you have a greater societal responsibility*.” In this understanding, nurses were framed as having special skills both useable and needed outside the health care system. With this understanding, nurses were seen as having a special moral obligation to provide guidance and protection to society extending beyond their work‐life in the health care system. A nurse explained:

As a nurse, you have to take the lead and lead the way (…). You have to be a kind of role model. You also have to be the one that the individuals outside the health care system can turn to and ask: “what shall we do? How are we going to solve this? What do you recommend?” So you have kind of been a nurse 24‐hours a day. (…) I felt like I had to speak on behalf of the entire health care system and say: “Remember to keep your distance and remember to wash and sanitize your hands.”

For the nurses who expressed an ethical demand emerging from the protection of society, failure to answer this ethical demand could come from many places. As demonstrated earlier, it could come from not acknowledging one's duty as a nurse to step up for society, its health professionals, and its patients. However, failure was also seen as something that could come from personal neglect and from not exercising proper caution in everyday life. As one example, becoming sick with COVID‐19 was identified as a threat among the nurses, albeit a threat that to some extent could be managed by making sacrifices on a personal level for the greater good. A nurse told during an interview how she felt that becoming sick from hanging out with friends was to neglect her duties. Therefore, she had chosen to stay isolated from social relations as much as possible.

I just couldn't bring myself to do it, and then afterward have to say to my superior: “well, I have to be on sick leave due to coronavirus, because I have been out enjoying myself with my friends.”

Even when COVID‐19 restrictions eased considerably during the fall of 2020, another nurse told how staying away from the gym for her had been the only acceptable choice in consideration for her patients: “*I don't go to the gym anymore because of the risk of picking up the virus and infecting my patients. So, I terminated my membership*.”

Among the nurses', self‐discipline in work and private life to answer the ethical demand they felt emerging from society was a vital necessity. However, the material also revealed how self‐discipline was accompanied by a strict focus on disciplining other nurses. To ensure that others abstained from modes of work or private life that could be seen as working contradictory to the ethical demand from society was seen as very important. Among those nurses who found themselves faced by an ethical demand from society, intense expressions of condemnation were aimed towards those perceived as exercising an inadequate level of self‐discipline. As an example, one nurse said:

I have been very concerned about the behavior of my colleagues and their lack of focus on hygiene. They seemed indifferent and have not cared about hygiene (…). I am convinced that I sanitize my hands far more often than most colleagues.

Other nurses emphasized how self‐discipline should be seen as extending into the personal life of nurses. Leading a life outside the hospital that could potentially jeopardize patients, colleagues, and society, in general, was generally seen as a violation of one's obligations as a nurse and thus severely frowned upon. As an example, one nurse added:

Fortunately, the curve has turned in Denmark, but it can quickly turn back around again (.) It is required that you always be aware of keeping your distance and performing proper hand hygiene, and so on. This makes it especially problematic with the young nurses. They went to Stockholm (Stockholm had high infection numbers at the time) and did not quarantine before going to work again.

These nurses expressed a need to self‐regulate their practices at work and home.

### 3.3 Nursing and personal ethical demands

Our analysis found that most nurses experienced extensive ethical demands emerging from their private sphere. The analysis revealed that many nurses experienced an ever‐present fear of infecting their family and friends. The nurses seemed particularly concerned about infecting vulnerable family members. These nurses understood the needed response to the pandemic as a need to prevent exposing their loved ones to danger due to their nursing profession. In a free‐text comment, a nurse stated: “*It's hard to constantly fear infecting my relatives and other people in my private life*.” Another nurse said:

I was scared. I was so scared! I just wanted to run away screaming. I was afraid of the fact that we didn't know how it would turn out in Denmark. Afraid I'd get sick, and I'd infect my child. I'm a single mom, so I was afraid of how it would affect him and me. I have constantly been affected by the fact that I'm the only one taking care of him.

For the safety of vulnerable family members, the nurses would deselect physical contact with vulnerable friends and family members. Sometimes with significant personal consequences as a result.

A nurse wrote:

It has been challenging to find yourself at risk of infection when your nearest relative is declared terminal. I've been so afraid of infecting her. For that reason, I have lost valuable final time with my mother.

For a few nurses, the ethical demand emerging from their families completely outweighed any societal, ethical demands. A nurse, who had refused to participate in the COVID‐19 contingency team due to the safety of her family, remarked:

I was ready to throw away my authorization as a nurse because I would simply not be part of a system that works that way (…) Well, as I said, I've resigned from The Danish Nursing Council. If it becomes necessary, I'll also throw away my authorization. My private life is more important than my work.

Another nurse described how considerations for her child with disabilities without a doubt outweighed her responsibilities as a nurse:

I've chosen to resign as a nurse because I'm also home‐caring for a child with a disability. I felt very uncomfortable because I could be bringing the infection home to my child. It was a tough decision, and I miss my colleagues and my job. But the little girl's safety is more important.

The material revealed that when nurses respond to personal ethical demands over societal, ethical demands, it prompted tensions. Thus, when multiple ethical demands are entangled, feelings of shame and guilt arose as a result. A nurse who had refused to join the corona contingency team explained how this decision caused her to feel guilty and to reconsider if she had ought to join her colleagues in the corona contingency team:

I had colleagues who broke down. Either because they either were about to be deployed to the corona‐ward or because they simply wanted to get away from the corona‐ward. It was awful to watch, and I felt so guilty because I had refused to be voluntarily deployed to the corona‐ward. So, I felt guilty towards my colleagues who had been sent over there. Should I have accepted to go there? Stepping up and participating is part of the nursing deed. As a nurse, you have to step up. To me, this was the hardest part.

### 3.4 Nursing and conflicting ethical demands

A consistent finding in the analysis was the experience of conflicting ethical demands. The nurses felt torn between responding to both societal and personal demands, which often were experienced as irreconcilable demands; the nurses could not fulfill their responsibilities as a nurse while securing the safety of their families. One nurse uttered:

At the same time, a part of me thought it was unsafe. It was unknown, and my children were apprehensive about whether I became infected at some point. Do I have to consider what will happen to my kids if something happens to me? So, on the one hand, I wanted to say that I didn't want to have anything to do with this, and the other part of me, the professional part, was ready for action, so to speak.

Another nurse explained:

Being a nurse at this time is full of dilemmas, especially if your close family member is at risk. This can create insecurity and lead to many sacrifices, such as less family time. As a nurse, you cannot make a corona‐closed circle with your family because you can never exclude patients and colleagues. These days, as a nurse, you can feel that your freedom as a human being is robbed from you, even if you are happy about your job.

Across interviews, we found that many nurses deal with these conflicting ethical demands by changing daily life routines and taking a wide range of practical precautions to respond to both types of ethical demands. Nurses tried their utmost to fulfill their duties as nurses during a pandemic while ensuring the safety of their families. One nurse said:

There is no doubt that it has been a strain along the way in relation to have to manage the risk of infection. I've been hysterical, used sanitizer and changed clothes before I drove home, and then changed clothes again before I went into the house. I divided the whole house into zones and felt like I had to do all kinds of things, crazy in fact.

The trouble navigating conflicting ethical demands gave rise to reflections on whether the work is worth it. One nurse stated in a free‐text comment:

The uncertainty about—if you were to get successive complications such as lung disease and maybe it wouldn't be recognized as a work‐related injury. It makes you think. Am I willing to expose myself to this? In my family, we've discussed whether a job, in general, is worth exposing one to such risks?

A recurrent theme in the interviews was the distinct emotional consequences of navigating conflicting ethical demands. Thus, many nurses experienced that all their efforts to ensure the safety of their family while caring for their work as a nurse had significant personal consequences such as loneliness, stress, deprivation, and loss.

Well, I've become kind of socio‐phobic, I think you could call it. I don't eat lunch with my colleagues, I don't go out for coffee. I keep to myself. A small group of colleagues talked about having a small Christmas party. I simply don't feel like it, and I am usually a very extrovert party animal, so it's very different… I've changed during this process. And then I can see this kind of social anxiety that I've developed afterward becauseI don't want to meet up with our friends or family or anything. I just don't want to. I simply can't embrace it now, so it's had an exaggerated effect.

## 4 DISCUSSION

We infer that the arrival of the COVID‐19 pandemic in Denmark may be understood as a moral breakdown raising very different ethical demands for nurses working in Danish public hospitals.

Several studies highlight how nurses' experienced, ethical challenges during the COVID‐19 pandemic (Gebreheat & Teame, 2021; Hossain & Clatty, 2021; Marey‐Sarwan et al., 2021; McConnell, 2020; Morley et al., 2020). It is well established that lack of personal protective equipment and prioritization of resources constitute the starting point of nurses' ethical challenges (Morley et al., 2020). When a pandemic evolves, the risk of infection and severity of the disease increases, health care resources become scarce and, therefore, subject to prioritization (ibid). Increasing demands for treating patients under such circumstances may put nurses at increased risk of contracting a severe disease while caring for patients and ultimately increase the risk of spreading the disease to their significant relations (Sperling, 2021). Managing the dichotomy of providing proper care for patients at one end while still protecting one's significant relations from potential diseases is common to the field of nursing care (Malm et al., 2008; McConnell, 2020). According to McConnell and colleagues (2020), such balancing between a duty to treat and duty to protect one's family from potential infections may usually be managed rather unreflexively by health professionals in routine care.

Nevertheless, as we have demonstrated, when the danger posed by the pandemic increases, so too do the stakes, making the dichotomy subject of reflection and ethical deliberation multiple. Our findings resonate with Hossain and Clatty's (2021) account of nurses' moral injury during the COVI‐19 pandemic and studies by Marey‐Sarwan and colleagues (2021) of nurses' experiences during the pandemic. They also underline how a pandemic onset may act as an engine for reflection, raising new ethical dilemmas between ideas of being a good nurse with a professional obligation to treat in times of COVID‐19 risk and crises and being a responsible partner protecting one's significant family relations from risks of COVID‐19 disease.

Following Zigon (2007), when morality breaks down due to crisis situations, ethical demands become raised, subject of reflection and deliberation ultimately demands the development of what may be seen as appropriate ethical management strategies emerge accordingly. When seen in light of the COVID‐19 pandemic, existing studies find that when faced with new ethical demands of family versus duty to treat, the risky conditions created by a pandemic may prompt nurses to prioritize protection one's family, thereby overriding the duty to treat (McConnell, 2020). Our analysis displays similar results. Our study found one group of Danish nurses who framed protection of their social lives and significant relations outside the health care system as the most salient ethical demand raised by the arrival of the COVID‐19 pandemic in Denmark. We here demonstrated how these perceived their role as nurses during the pandemic in terms of accommodation, molding their nursing role to accommodate the needs of their significant relations best. For some nurses, this even translated into a willingness to leave the profession if necessary to respond to this ethical demand.

Contrastingly we also found a group of Danish nurses who saw their primary ethical demand being a duty to treat patients at all costs. We here demonstrated how this perceived duty to treat a patient was framed in ideas of nurses being a vital pillar in society with an inherent responsibility towards protecting society and its citizens. Ethical moments bring together multiple moral aspects that inform how ethical demands become articulated and subject to ethical action (Zigon, 2009). According to Zigon (2008), these aspects are drawn from moral spheres, that is, institutional, habitual, and, importantly, public discourses such as media discourses that may exert an influential role in the ethical moment. This topic of media framing nurses as a supererogatory profession, a vital pillar in society with salient responsibilities to society in the time of COVID‐19 (nurse‐as‐hero), has been raised in several recent papers (Boulton et al., 2021; Cox, 2020; Halberg et al., 2021; McAllister et al., 2020; Stokes‐Parish et al., 2020). Common to these studies is a pronounced distancing found among nurses towards the media and the public's appointment of them as heroes with special societal obligations. According to Cox and colleagues (2020), the media and enormous public praising healthcare workers as heroes can have potentially adverse consequences (Cox, 2020). More studies infer that nurses resist the hero narrative due to its inherent predefined characteristics, its tendency to stifle meaningful discussion about the extent of the nurses' duty, and it fails to acknowledge the importance of reciprocity (Cox, 2020; Halberg et al., 2021; McAllister et al., 2020). Our findings tell a somewhat different story. Our material displayed prominent examples of ethical demands that frame nurses in roles with a special obligatory duty to set patients' and society's needs above personal needs. These nurses' views on a nurse's responsibilities and obligations in this sense strongly resemble the image that the media and the public paint of nurses as self‐sacrificing heroes who neglect their health for the sake of the greater good. For example, more nurses in our study supported the idea of nurses as “model citizens,” which is in line with Mohammed and coworkers' interpretation of the main elements of the nurse‐as‐hero discourse (Mohammed et al., 2021).

Moreover, when responding, they chose to acknowledge their duties to society. They also perceived it as necessary to self‐discipline professional and social life for the good of society to respond successfully to this ethical demand. Though as seen, our material also displays examples of nurses actively rejecting the hero‐narrative favoring family over society. Our results nevertheless indicate that the unilateral dissociations among our nurses with the nurse‐as‐hero narrative (as something being forced upon them) otherwise found in several existing papers could benefit from further nuances (Cox, 2020; Halberg et al., 2021; McAllister et al., 2020). Not only did some of the nurses acknowledge an ethical demand towards society, but they also actively chose to enforce it in practice via ethical self‐discipline strategies favoring consideration towards society over consideration of family. These findings suggest a need to approach the nurse‐as‐hero discussion more cautiously and acknowledge that nurses are a heterogeneous group with widely differing views on the boundaries of their ethical demands.

Though certain moral spheres may at times dominate and articulate more clear ethical demands demarcating what a suitable ethical response may be, according to Zigon (2008, 2009), humans are not automatons, and societies in which they inhibit are complex. Therefore, human lives most often unfold within a complex intersection of moral spheres, providing possibilities to overcome moral questioning, but they may also raise sometimes conflicting or competing for ethical demands (Zigon 2009). This strongly resonates with our analysis demonstrating how a substantial group of participants perceived their nursing role during the COVID‐19 pandemic in response to entangled ethical demands orientated towards society and their social lives and relations outside the health care system. Quoting French philosopher Alain Bardiou (2001), Zigon (2007) notes that ethical work may be described as a process of “keep going,” that no matter the odds, one must work to resolve the ethical demands raised. Here, the multiple moral spheres of society may provide room for creativity and maneuverability, ultimately providing a means for achieving ethical resolution (Zigon, 2009). Our study suggests that nurses were still struggling in the negotiation process with no explicit expressions of a creative resolution being insight. Contrastingly, the nurses felt they were being kept in the intricate ethical moment. This was experienced as a conflict giving rise to feelings of distress of a moral kind. This finding resonates with Rushton's (2017) account of moral distress among nurses. According to Rushton, moral distress occurs when “one recognizes one's moral responsibility in a situation; evaluates the various courses of action; and identifies, in accordance with one's beliefs, the morally correct decision—but is then prevented from following through” (Rushton, 2017). Rushton further argues that unresolved moral distress has been correlated with burnout and long‐term consequences such as emotional exhaustion, depersonalization, feeling disengagement, numbness, and diminished moral sensitivity (Rushton, 2017). Such consequences are retrieved in our material, as our analysis indicates that the entanglement of ethical demands was perceived as demanding flexibility by the nurses, which often prompted feelings of loneliness, stress, and deprivation. Nurses' experiences of entangled ethical demands may therefore contribute to the massive distress documented among this group of healthcare professionals during the pandemic (Luo et al., 2020; Pappa et al., 2020; Sanghera et al., 2020; Vizheh et al., 2020).

### 4.1 Implications for nursing

Our study may have implications for formalizing the ongoing emotional, psychological, and practice support of nurses during (and after) the pandemic. As the pandemic continues to pose severe challenges for healthcare professionals worldwide, it is critical to consider how to alleviate the emotional, psychological, and ethical challenges nurses face in their everyday practice. Keeping in mind that nurses constitute the biggest group of health care professionals working in the context of the COVID‐19 pandemic (James Buchan, 2020), securing their safety and mental well‐being is essential. Therefore, in healthcare organizations' providing practical measures to address the ethical distress experienced by their workers, being suspended in conflicting ethical demands is necessary.

## 5 LIMITATIONS

There are some limitations to our study. Due to the pandemic, all interviews were conducted from a distance using the telephone, and the absence of visual cues may have precipitated a loss of contextual and nonverbal data. In addition, the self‐selection sampling method used in the survey may have limited perspectives revealed in the current study's findings because there is likely to be a degree of self‐selection bias. For example, the decision to participate in the survey may reflect some inherent bias in the traits of the participants—for example, nurses with strong opinions or negative experiences. However, both the empirical interview and free text material revealed broad and nuanced perspectives on how ethical challenges related to “being a nurse” during the COVID‐19 pandemic were experienced, and all participants shared both positive and negative experiences. In addition, the timing of the data collection is also a limitation to the study. We collected the data at the beginning of the pandemic, and given how much the pandemic has evolved since then, having interviewed the nurses later on during the pandemic would add another layer to understanding ethical demands. Finally, we only included nurses working in hospitals. Including empirical material based on nurses working outside the hospitals would have added a more nuanced understanding of ethical challenges experienced by nurses across healthcare settings. Theoretically, we applied Zigon's (2007) notion of moral breakdown to understand the perceptions and trajectories pursued by the nurses in our study during the COVID‐19 pandemic. By using Zigon's framework, we could engage in discussions of morality, nursing, and COVID19 on a theoretically conceptual level. According to Zigon (2007), moral breakdowns and their responses are always products of entangled socio‐cultural and institutionalized moral processes inherent to human social life. Our paper, however, only provides knowledge about a particular moment of ethical response (Zigon, 2007, 2008). We, therefore, acknowledge that our paper does not provide knowledge regarding the social‐cultural origins of the ethical demands presented in our analysis. Nor does our paper provide knowledge concerning the result of the ethical work of nurses and any forms of ethical resolution after the study had ended.

## 6 CONCLUSION

This study has shown how the rapid onset of the pandemic can be understood as a moral breakdown raising ethical demands for nurses working in hospitals during COVID‐19. Three different ethical demands were identified, highlighting the differences among the nurses when it came to which ethical demands were perceived most salient. One of the more significant findings to emerge from this study is that a substantial group of participants perceived their nursing role during the pandemic in terms of response to entangled ethical demands. For more nurses, challenges in dealing with these entangled ethical demands prompted a feeling of loneliness, stress, and deprivation.

## CONFLICT OF INTEREST

The authors declare no conflict of interest.

## Data Availability

The data that support the findings of this study are available from the corresponding author upon reasonable request.
